# Zinc-Doped Copper Oxide Nanocomposites Inhibit the Growth of Pancreatic Cancer by Inducing Autophagy Through AMPK/mTOR Pathway

**DOI:** 10.3389/fphar.2019.00319

**Published:** 2019-04-02

**Authors:** Xiao Li, Huanli Xu, Cong Li, Gan Qiao, Ammad Ahmad Farooqi, Aharon Gedanken, Xiaohui Liu, Xiukun Lin

**Affiliations:** ^1^Department of Pharmacology, School of Basic Medical Sciences, Capital Medical University, Beijing, China; ^2^Laboratory for Translational Oncology and Personalized Medicine, Rashid Latif Medical College (RLMC), Lahore, Pakistan; ^3^Center for Advanced Materials and Nanotechnology, Bar-Ilan University, Ramat Gan, Israel

**Keywords:** zinc doped copper oxide nanocomposites, pancreatic cancer, *in vivo* activity, autophagy, AMPK

## Abstract

Zinc doped copper oxide nanocomposites (Zn-CuO NPs) is a novel doped metal nanomaterial synthesized by our group using the sonochemical method. Our previous studies have shown that Zn-CuO NPs could inhibit cancer cell proliferation by inducing apoptosis via ROS-mediated pathway. In the present study, we studied the anticancer effect of Zn-CuO NPs on human pancreatic cancer cells. MTS assay revealed that Zn-CuO NPs was able to inhibit cancer cell growth. TEM, flow cytometry and fluorescence microscope analysis showed that Zn-CuO NPs induced autophagy significantly; the number of autophagosomes increased obviously in cells treated with Zn-CuO NPs. Western blot analysis revealed that treatment with the NPs resulted in activation of AMPK/mTOR pathway in both AsPC-1 and MIA Paca-2 cells in dose dependent manners. Moreover, in the presence of AMPK activator AMPKinone, the protein level of p-AMPK, p-ULK1, Beclin-1 and LC3-II/LC3-I increased, while the protein expression of p-AMPK, p-ULK1, Beclin-1 and LC3-II/LC3-I decreased in the presence of AMPK inhibitor Compound C. *In vivo* study using xenograft mice revealed that Zn-CuO NPs significantly inhibited tumor growth with low toxicity. Our study confirms that Zn-CuO NPs inhibit the tumor growth both *in vitro* and *in vivo* for pancreatic cancer. AMPK/mTOR pathway plays an important role in the NPs induced inhibition of tumor growth.

## Introduction

Pancreatic cancer is one of the most fatal diseases around the world with a 5 year survival of approximately 6% (Balachandran et al., 2017; Knudsen et al., 2018). Although some advances have been made in the treatment of pancreatic cancer by surgery, radiation therapy and chemotherapy in recent years, the 5 year survival rate still remains low (Krempien and Roeder, 2017; Tempero et al., 2017). Therefore, there is an urgent need to explore high-efficient and low-toxic drugs for the treatment of pancreatic cancer.

Metal oxide NPs exhibit unique physical and chemical properties due to their limited size and large surface area. Metal oxide NPs have been widely used in medicine, catalysis, sensors, environmental remediation, personal care products, cosmetics, etc. (Magdalane et al., 2016; Saleem et al., 2017; Su et al., 2017). In the field of medicine, metal oxide NPs show great prospects in some fields, including drug delivery, diagnostics, or regenerative medicine (Rasmussen et al., 2010; Shi et al., 2017). Metal oxide NPs, such as ZnO, CuO, and Fe_3_O_4_ NPs have been reported to cause genotoxicity, mitochondrial dysfunction and induce cell apoptosis and autophagy in many cancer cell lines (Baek and An, 2011; Chang et al., 2012; Zhao et al., 2013; Dizaj et al., 2014; Khosravi-Katuli et al., 2018). Zn-CuO NPs, (Cu_0.89_ Zn_0.11_O), is a novel doped metal nanomaterial synthesized by our group using the sonochemical method (Malka et al., 2013). Our previous study showed that Zn-CuO NPs could inhibit cancer cell proliferation by inducing apoptosis via ROS-mediated pathway (Yuan et al., 2016). We also found that the nanomaterials were able to inhibit the glioma both *in vitro* and *in vivo* (Wu et al., 2018).

Autophagy, a steady-state cell degradation process used to remove damaged or unnecessary organelles and proteins, plays an important role in cancer development (Isaka et al., 2017). Recent study has shown that autophagy is also involved in metal oxide NPs induced cancer cell apoptosis (Alinovi et al., 2017; Kermanizadeh et al., 2017). Iron oxide NPs can selectively induce autophagy in cancer cells, thereby significantly killing cancer cells without toxic effects on normal cells (Khan et al., 2012). Zinc oxide NPs are reported to induce cell death in macrophage cells through enhancement of autophagy via PI3K/Akt/mTOR inhibition (Roy et al., 2014). Copper oxide NPs (CuO NPs) are proved to induce autophagic cell death in A549 cells (Sun et al., 2012). Our recent study also reveals that Zn-CuO NPs induced autophagy in pancreatic and hepatocellular carcinoma (Xu et al., 2018). However, the signal pathway of Zn-CuO NPs induced autophagy remains unknown.

AMPK, a key energy sensor, regulates energy homeostasis and metabolic stress by controlling several homeostatic state, including autophagy and protein degradation (Kim et al., 2013; Li et al., 2013). AMPK mediates the activity of mTORC1 via phosphorylating Raptor and TSC2, two negative regulator of mTORC1, to induce autophagy (Alers et al., 2012). Meanwhile, AMPK could directly interact with Ulk1 and positively regulate its activity through AMPK-dependent phosphorylation, further expanding the range of possibilities for AMPK induced autophagic process (Papinski and Kraft, 2016). Studies have shown that a lot of anticancer agents display activity via activating AMPK/mTOR signaling pathway. Salen-Mn, a synthetic reagent, was reported to inhibit the growth of human prostate cancer cells through APMK pathway; treatment of the cancer cells with salen-Mn resulted in higher expression of LC3I/II (Tang et al., 2017). Rographolide, the main bioactive ingredients of *Andrographis paniculata*, is able to induce apoptosis of human nasopharyngeal carcinoma cells through LKB1-AMPK-dependent signaling pathway. Oleuropein aglycone, the major phenolic component of extra virgin olive oil, also induces autophagy via the AMPK/mTOR signaling pathway (Rigacci et al., 2015). In this study, we examined the anti-tumor effects of Zn-CuO NPs on pancreatic cancer both *in vitro* and *in vivo*. Our results showed that Zn-CuO NPs exhibited potent anti-tumor effect on pancreatic cancer. AMPK/mTOR pathway plays an important role in Zn-CuO NPs-induced inhibition of human pancreatic cancer cells.

## Materials and Methods

### Cell Lines and Cell Cultures

Human cancer cell lines, including human liver cancer cells (HepG2), human colon cancer cells (HT-29), human pancreatic cells (AsPC-1, PANC-1, BxPC-3, and MIA PaCa-2) and astrocyte cells were purchased from the American Type Culture Collection (ATCC, MD, United States). Human pancreatic MIA PaCa-2 and AsPC-1 cancer cells were cultured in RPMI 1640 medium (Gibco, CA, United States) supplemented with 10% fetal bovine serum (FBS; Gibco, CA, United States), and all of the other cells were cultured in high glucose Dulbecco’s Modifed Eagle’s Medium (DMEM; Gibco, CA, United States) supplemented with 10% FBS in a humidified atmosphere at 37°C with 5% CO_2_.

### Chemicals and Reagents

Cell culture medium, DMEM, RPMI-1640, trypsin, phosphate-buffered saline (PBS), and FBS were purchased from Gibco (CA, United States). DMSO, 3-Methyladenine (3-MA), Compound C (HY-13418), and AMPKinone (HY-12831) were products of MedChem Express (NJ, United States). Antibodies against AMPK, p-AMPK, mTOR, p-mTOR, ULK, p-ULK, Beclin-1, and LC3I/II were obtained from Cell Signaling Technology (Danvers, MA, United States). Horseradish peroxidase-conjugated secondary antibody (IgG goat anti-rabbit or anti-mouse) and beta -tubulin antibody were purchased from CWBIO. BCA protein assay kit was product of Beyotime (Shanghai, China). MTS assay kit was purchased from Promega (Promega, WI, United States). All other chemicals used were the highest purity obtained from commercial sources.

### 3-(4,5-Dimethylthiazol-2-yl)-5-(3-Carboxymethoxyphenyl)-2-(4-Sulfophenyl)-2H-Tetrazoli-um (MTS) Assay

Drug effects on cell viability were analyzed using MTS assay kit (Promega, WI, United States) as per the manufacture’s protocol. Briefly, cancer cells (3 × 10^3^ cells/well) were seeded in flat-bottomed 96-well plates. After overnight incubation, the cells were treated without or with certain concentrations (5, 10, 20, 40, 60, 80, 120, and 160 μg/ml) of Zn-CuO NPs for 24 h, and MTS was added to each well and incubated for another 1 h. Then, the absorbance values were detected with an ELISA reader at a wavelength of 490 nm. Data were expressed as mean ± SD; *n* ≥ 3 per group for all of the studies. All of the experiments were performed in 3 independent times. The half maximal inhibitory concentration (IC_50_) was defined as the concentration resulting in 50% cell growth reduction compared with untreated control cells. IC_50_ was analyzed using Graphpad Prism 5.0.

### Autophagy Detection by Transmission Electron Microscopy (TEM) and Flow Cytometry

AsPC-1 and MIA PaCa-2 cells were treated without or with Zn-CuO NPs (40.0 and 50.0 μg/ml, respectively) for 24 h, fixed with 2.5% glutaraldehyde, and washed three times with 0.1 M phosphate buffered saline (PBS, pH7.2). Then, cells were fixed with 1% osmium acid for 1–2 h and washed with 0.1 M PBS buffer for three times. The samples were dehydrated with increasing concentration of ethanol (30, 50, 70, 80, 90, and 100%). Samples were stained by cellulose acetate and lead nitrate solution and then observed by transmission electron microscope (Jeol, Japan).

Autophagy flow was analyzed as described previously (Tian et al., 2018). Briefly, AsPC-1 and MIA PaCa-2 cells (1 × 10^5^) were plated in 6-well plates. After incubation for 24 h, cells were infected with adenovirus harboring tandem fluorescent mRFP-GFP-LC3 (Hanbio Inc., China) at 1,000 multiplicity of infection. After incubation for another 24 h, Zn-CuO NPs were added with the concentration of 40.0 and 50.0 μg/ml and incubated for 24 h. The autophagy flow was observed under a fluorescence microscope (Nikon Eclipse E800).

Quantification of autophagy was determined using CYTO-ID autophagy detection kit 2.0 (Enzo life sciences, United States) as per the manufacturer’s instructions. Briefly, cells (1 × 10^5^) were plated in 6-well plates. After incubation for 24 h, cells were treated without or with Zn-CuO NPs (10.0, 20.0, 40.0 and 12.5, 25.0, 50.0 μg/ml) for 24 h and then collected by centrifugation at 1,000 rpm for 5 min. After washing 3 times with 1 × buffer, the cells were resuspended in 1 × buffer of the kit and then stained with CYTO-ID for 30 min at 37°C in the dark. The value of fluorescence intensity of autophagosomes was detected using a flow cytometer (BD LSRFortessa SORP, United States).

### Western Blot Analysis

Cancer cells were treated without or with Zn-CuO NPs (10.0, 20.0, 40.0 and 12.5, 25.0, 50.0 μg/ml, respectively). The concentrations of AMPKinone and Compound C were 10 and 5 μM, respectively. After incubation for 24 h, cells were washed twice with ice-cold PBS and lysed using lysis buffer (Beyotime, Shanghai, China). The protein supernatant was collected by centrifugation at 12,000 rpm for 15 min at 4°C. Protein concentrations were determined using the BCA protein assay kit (Thermo Scientific, CA, United States). Equal amounts of protein (20 μg/sample) was resolved by 8–12% SDS-PAGE and transferred onto 0.45 mm PVDF membranes (Millipore, NY, United States). The membranes were blocked with 5% skim milk in TBS-T buffer [10 mmol/L Tris (pH 7.4), 150 mmol/L NaCl, and 0.1% Tween-20] at room temperature. After incubation for 1 h, the membrane was incubated with the primary antibodies including AMPK, phospho-AMPK, mTOR, phospho-mTOR, ULK, phospho-ULK, Beclin-1, LC3I/II (1:1000, Cell Signaling Technology), and β-tubulin (1:800, CWBIO) overnight. The membranes were washed 3 times with TBS-T buffer, and then incubated with horseradish peroxidase (HRP)-conjugated secondary antibodies (IgG goat anti-rabbit or anti-mouse; 1:800; CWBIO) for half an hour at room temperature. The protein bands were visualized with Super Signal West Dura Extended Duration Substrate (ThermoScientific, United States). The gray intensities of the bands were analyzed using Image J software. All data were obtained by at least three independent experiments.

### Anti-tumor Activity of Zn-CuO NPs in Tumor Bearing Nude Mice

Female BALB/c nude mice (4–6 weeks, 18–20 g) were randomly divided into the following four groups with five mice per group; control group (85% normal saline with 15% DMSO), Zn-CuO NPs group (5 mg/kg), Zn-CuO NPs group (10 mg/kg), and positive control (Cyclophosphamide, CTX, 50 mg/kg). Each mouse was inoculated subcutaneously with AsPC-1 cells (1 × 10^7^, 400 μl) in the right dorsal flank. After tumors grew up to approximately 100 mm^3^, the mice were treated by oral administration with Zn-CuO NPs and CTX daily for 14 days. Of note, the CTX was given totally two times. The tumor sizes and body weights were measured every 2 days using a vernier caliper, and the tumor volumes were calculated using the following formula; Tumor volume = tumor length × tumor width^2^ × 0.5. All animal experiments were performed through the ethical review by Animal Experiments and Experimental Animal Welfare Committee of Capital Medical University, in accordance with local institutional guidelines and ethics in Beijing, China (No. 1118032100240). The protocol was approved by local institutional guidelines and ethics in Beijing, China.

### Histological Examination and Immunohistochemistry Analysis

Mice were treated P.O. with Zn-CuO NPs, CTX as described above. At the end of the experiment, the mice were sacrificed and organs of the mice (heart, liver, spleen, lung, kidney, stomach, and pancreas) in each group were removed. Tumor tissues were peeled off and embedded in paraffin, and used for further histological examinations using hematoxylin-eosin staining. Paraffin sections (4–8 μm) were prepared and detected using immunostaining with the Ki-67 antibody (a proliferation index) (Shimoji et al., 2016). Images were scanned by Digital pathology slice scanner (3DHISTECH Ltd., Hungary).

### Statistical Analysis

Statistical analysis was performed using student *t*-test and the data were analyzed with Graphpad Prism 5.0 (Graphpad Software, San Diego, CA, United States). Data were considered to be statistically significant when ^∗^*p* < 0.05, ^∗∗^*p* < 0.01, ^∗∗∗^*p* < 0.001.

## Results

### Zn-CuO NPs Significantly Inhibited Cancer Cell Growth

We firstly examined the effect of Zn-CuO NPs on the viability of several cell lines by MTS assay. As shown in Table 1, Zn-CuO NPs were capable of inhibiting cancer cell growth significantly; the IC_50_ values of Zn-CuO NPs on HepG2, AsPC-1, MIA PaCa-2, BxPC-3, PANC-1, and HT-29 were 41.25 ± 2.13, 43.19 ± 3.18, 51.42 ± 4.11, 70.59 ± 3.86, 96.48 ± 5.97, and 168.71 ± 10.54 μg/ml, respectively. Since Zn-CuO NPs showed more potent effects against AsPC-1 and MIA PaCa-2 cells (Table 1), we used AsPC-1 and MIA Paca-2 cells for further studies.

**Table 1:** IC_50_ values of Zn-CuO NPs on different cell lines.

Cell lines	IC_50_ (μg/ml) mean ± SEM
HepG2	41.25 ± 2.13
AsPC-1	43.19 ± 3.18
MIA PaCa-2	51.42 ± 4.11
BXPC-3	70.59 ± 3.86
PANC-1	96.48 ± 5.97
HT29	168.71 ± 10.54
Astrocyte	>400


### Zn-CuO NPs Induced Autophagy in Cancer Cells

**FIGURE 1:**
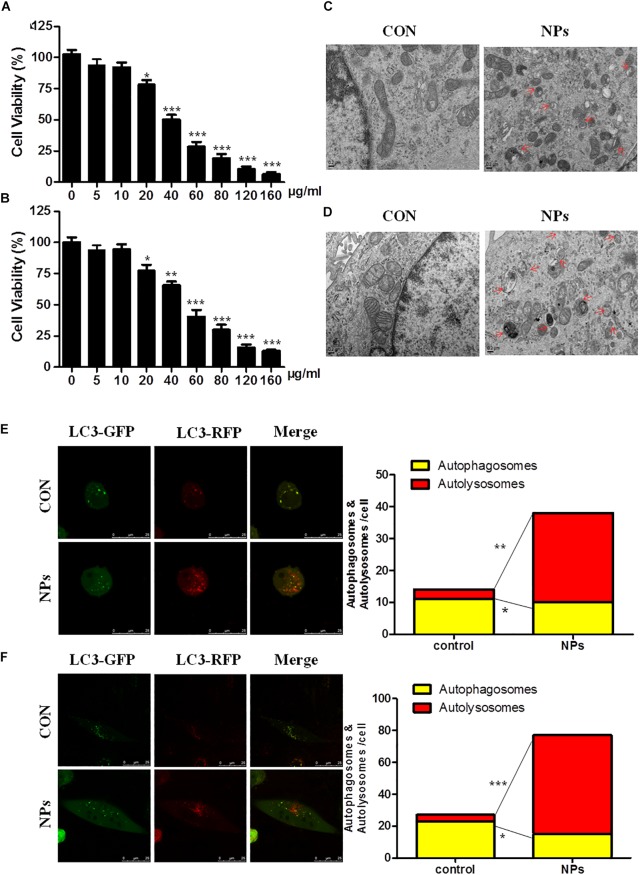
Zinc doped copper oxide nanocomposites induced autophagy in both AsPC-1 and MIA PaCa-2 cancer cells. AsPC-1 **(A)** and MIA PaCa-2 cells **(B)** were treated with certain concentrations of Zn-CuO NPs for 24 h, and the cell viability was determined by MTS assay (*t*-test, ^∗^*p* < 0.05, ^∗∗^*p* < 0.01, ^∗∗∗^*p* < 0.001). Autophagosomes in AsPC-1 and MIA PaCa-2 cells were observed by TEM. **(C,D)** Autophagy flow was determined by confocal fluorescence microscopy in AsPC-1 **(E)** and MIA PaCa-2 cancer cells **(F)**. Red puncta represents autolysosomes and yellow puncta represents autophagosomes (*t*-test, ^∗^*p* < 0.05, ^∗∗^*p* < 0.01, ^∗∗∗^*p* < 0.001 vs. control group).

The dose-dependent effect of Zn-CuO NPs on cancer cell growth was evaluated in both AsPC-1 and MIA Paca-2 cells (Figure 1A,B). The effects of Zn-CuO NPs on autophagy were studied using electron microscopy to detect intracellular autophagosomes. There are almost no autophagosomes in vehicle treated cells (Figure 1C, con). However, exposure of AsPC-1 and MIA PaCa-2 cells to Zn-CuO NPs (24 h) obviously increased the number of autophagosomes in the cytoplasm as indicated by the red arrow (Figure 1D, NPs). Next, autophagic flow assay was performed to further confirm Zn-CuO NPs-induced autophagy. As shown in Figure 1E,F, after infection with the mGFP-RFP-LC3 adenovirus (Hanbio Inc., China), both red and green puncta can be observed, showing the successful introduction of this adenovirus into the cells. There were more red puncta in cells treated with Zn-CuO NPs than those in cells in the control group; the number of autolysosomes increased from 4 and 16 in control to 28 and 62, respectively in AsPC-1 and MIA PaCa-2 cells treated with the nanoparticles. The results suggested that Zn-CuO NPs are able to induce autophagy in both AsPC-1 and MIA PaCa-2 cells.

**FIGURE 2:**
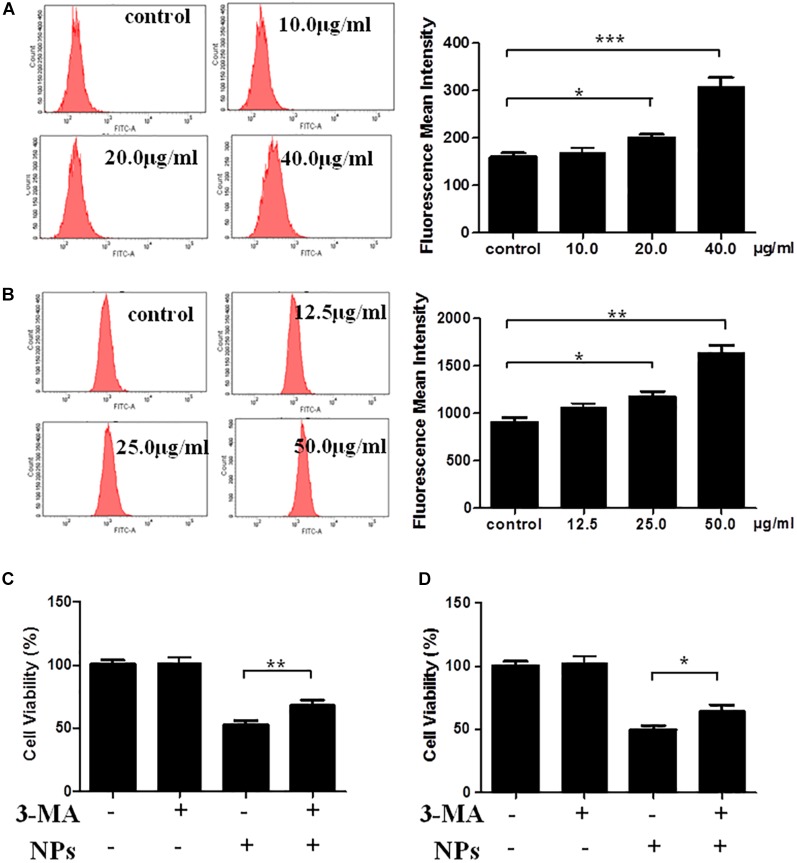
Autophagic inhibitor 3-MA enhanced the Zn-CuO NPs induced cell death in both AsPC-1 and MIA PaCa-2 cells. Quantification of autophagy was detected by flow cytometry for AsPC-1 **(A)** and MIA PaCa-2 cancer cells **(B)** as described in the Section “Materials and Methods.” Cells were treated with Zn-CuO NPs alone or the combination of Zn-CuO NPs and 3-MA. The relative cell viability in AsPC-1 **(C)** and MIA PaCa-2 **(D)** cancer cells was determined using MTS assay as described in the Section “Materials and Methods” (*t*-test, ^∗^*p* < 0.05, ^∗∗^*p* < 0.01, ^∗∗∗^*p* < 0.001).

The quantification of autophagy was performed using flow cytometry analysis. As shown in Figure 2A, Zn-CuO NPs treatment significantly increased the autophagy; the average relative value of fluorescence intensity increased from 159 to 170, 199, and 307, respectively after treated with Zn-CuO NPs at the concentration of 10, 20, and 40 μg/ml (*p* < 0.05). Similar results were also found in MIA PaCa-2 cells (Figure 2B). These results further confirmed that treatment with Zn-CuO NPs increased the autolysosome formation in both AsPC-1 and MIA PaCa-2 cells. To determine the properties of Zn-CuO NPs-induced autophagy, a small molecule inhibitor of autophagy, 3-MA, was used. As shown in Figure 2C,D, the cell viability was increased significantly in cells treated with the combination of Zn-CuO NPs and 3-MA, compared with that in AsPC-1 and MIA PaCa-2 cells treated with Zn-CuO NPs alone. This result suggested that inhibition of autophagy attenuated the cytotoxic activity of Zn-CuO NPs and the nanoparticles-induced autophagy contributed to their inhibitory effect on cancer cell growth.

### Zn-CuO NPs Activated AMPK/mTOR Pathway in Cancer Cells

**FIGURE 3:**
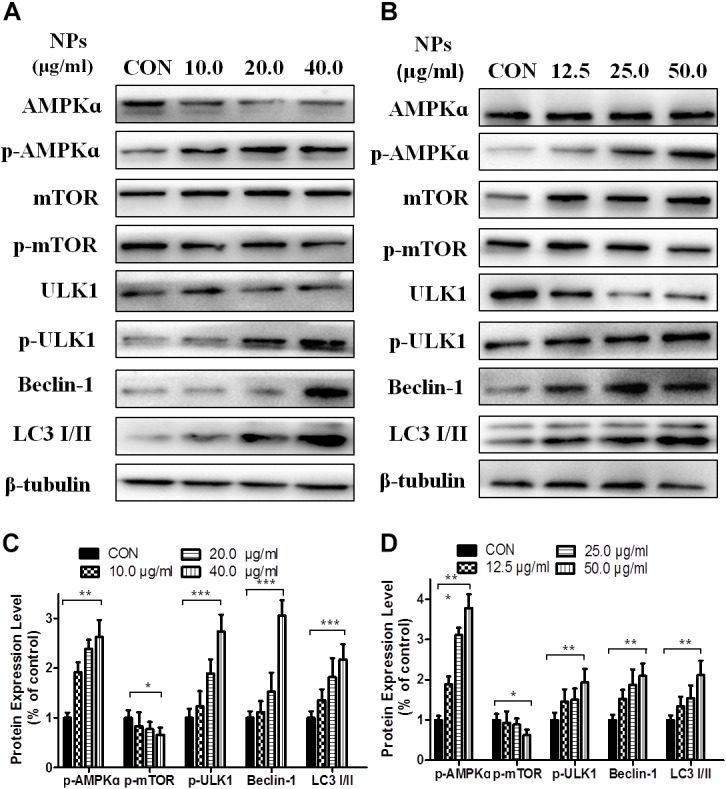
Zinc doped copper oxide nanocomposites induced autophagy via AMPK/mTOR pathway. The level of AMPK, p-AMPK, mTOR, p-mTOR, ULK1, p-ULK1, Beclin-1, and LC3-II/LC3-I were detected by Western Blot in AsPC-1 **(A)** and MIA PaCa-2 **(B)** cancer cells. **(C,D)** represented the quantitative results of relative autophagic gene expression (*t*-test, ^∗^*p* < 0.05, ^∗∗^*p* < 0.01, ^∗∗∗^*p* < 0.001).

Since AMPK pathway plays an important role in autophagy, we performed Western blot analysis to determine the effect of Zn-CuO NPs on AMPK/mTOR mediated autophagy, As shown in Figure 3, Zn-CuO NPs treatment increased the protein level of p-AMPK, p-ULK1, and Beclin-1, as well as the ratio of LC3-II/LC3-I, while down-regulated the mTOR phosphorylation in both AsPC-1 (Figure 3A) and MIA PaCa-2 cells (Figure 3B) in a dose-dependent manner. The results indicated that AMPK/ULK pathway played an important role in Zn-CuO NPs induced cell autophagy.

**FIGURE 4:**
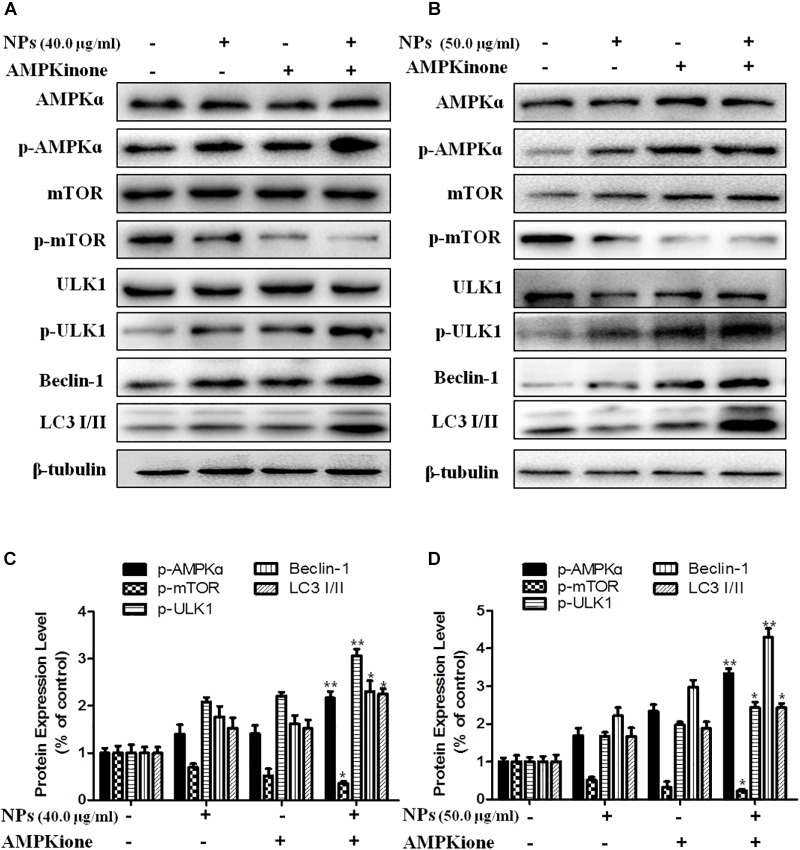
AMPK/ULK pathway played an important role in Zn-CuO NPs-induced autophagy. Cells were treated with Zn-CuO NPs or the combination of Zn-CuO NPs and AMPKinone. The level of proteins related with AMPK/ULK pathway was determined using Western blot in AsPC-1 **(A)** and MIA PaCa-2 cancer cells **(B)**. **(C,D)** represented the quantitative results of relative autophagic gene expression (*t*-test, ^∗^*p* < 0.05, ^∗∗^*p* < 0.01, ^∗∗∗^*p* < 0.001 vs. Zn-CuO NPs group).

**FIGURE 5:**
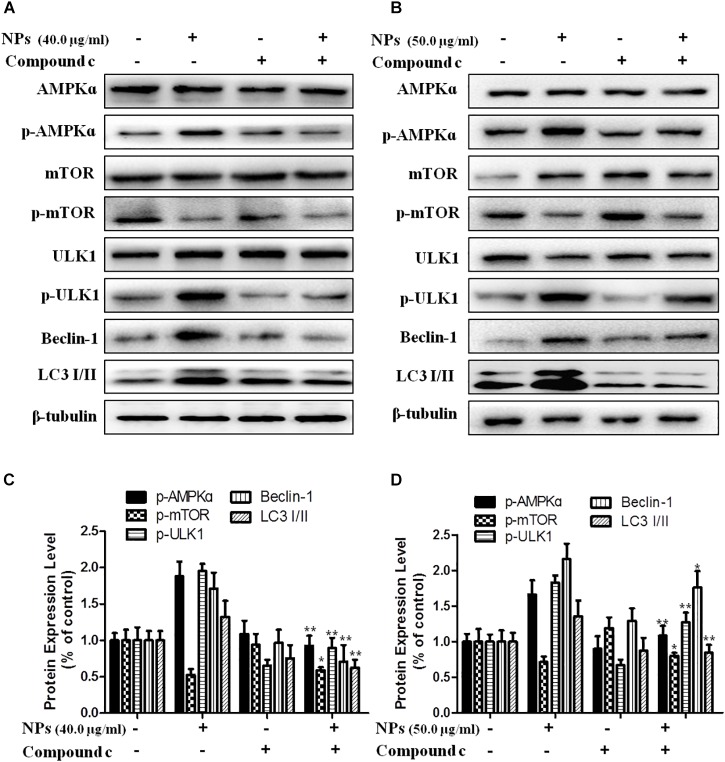
AMPK/ULK pathway played an important role in Zn-CuO NPs-induced autophagy. Treatment of cancer cells with Zn-CuO NPs or the combination of Zn-CuO NPs and Compound C, the level of proteins related with AMPK/ULK pathway was determined using Western blot in AsPC-1 **(A)** and MIA PaCa-2 cancer cells **(B)**. **(C,D)** represented the quantitative results of relative autophagic gene expression (*t*-test, ^∗^*p* < 0.05, ^∗∗^*p* < 0.01, ^∗∗∗^*p* < 0.001, vs. Zn-CuO NPs group).

In order to further verify that Zn-CuO NPs triggered autophagy was mediated by activating AMPK pathway, we determined the gene expression related with AMPK-mediated autophagic pathway in the presence of Compound C (an AMPK inhibitor) and AMPKinone (an AMPK activator) using Western blot analysis, and the results showed that the protein level of p-AMPK, p-ULK1, Beclin-1, and LC3-II/LC3-I was increased, while the mTOR phosphorylation was diminished significantly in cells treated with the combination of Zn-CuO NPs and AMPKinone (10 μM), compared with that in cells treated with Zn-CuO NPs alone (Figure 4). Additionally, treatment with the combination of Zn-CuO NPs and Compound C, the protein level of p-AMPK, p-ULK1, Beclin-1, and LC3-II/LC3-I was declined compared with that in cells treated with Zn-CuO NPs alone (Figure 5). The results confirmed that Zn-CuO NPs was able to induce autophagy through AMPK/mTOR pathway in both AsPC-1 and MIA PaCa-2 cells.

### Zn-CuO NPs Inhibited the Tumor Growth in Xenograft Mice Model

**FIGURE 6:**
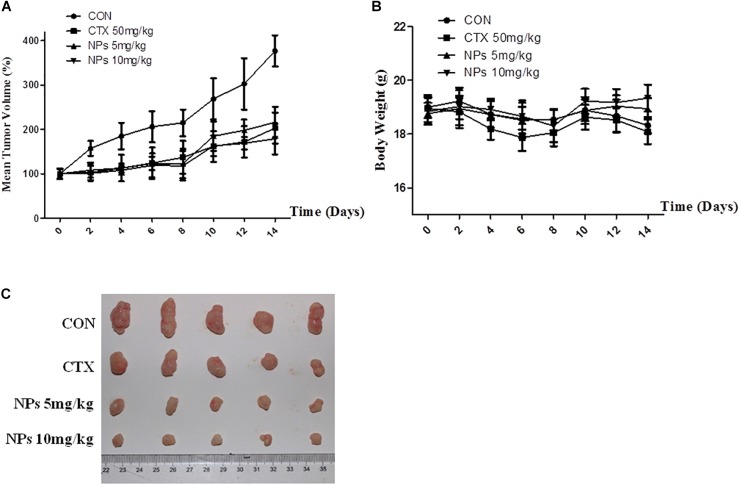
Zinc doped copper oxide nanocomposites inhibited tumor growth in AsPC-1 tumor xenograft mice model. BALB/c nude mice were inoculated with AsPC-1 cancer cells, and treated with Zn-CuO NPs P.O. and the tumor volume **(A)** and mouse body weight **(B)** were determined as described in the Section “Materials and Methods.” The collected tumor tissues of each group at the end of the experiment were photographed **(C)**.

AsPC-1 xenograft BALB/c nude mice were treated P.O. with Zn-CuO NPs at the dose of 5 and 10 mg/kg, respectively. As shown in Figure 6A and 6C, treatment with Zn-CuO NPs exhibited significant anti-tumor activity; the inhibitory rates were 46.1 and 52.6%, respectively when treating the mice with Zn-CuO NPs at a dose of 5 and 10 mg/kg, respectively (Figure 6A). Meanwhile, no significant body weight changes were observed in the Zn-CuO NPs and CTX treated groups (Figure 6B).

**FIGURE 7:**
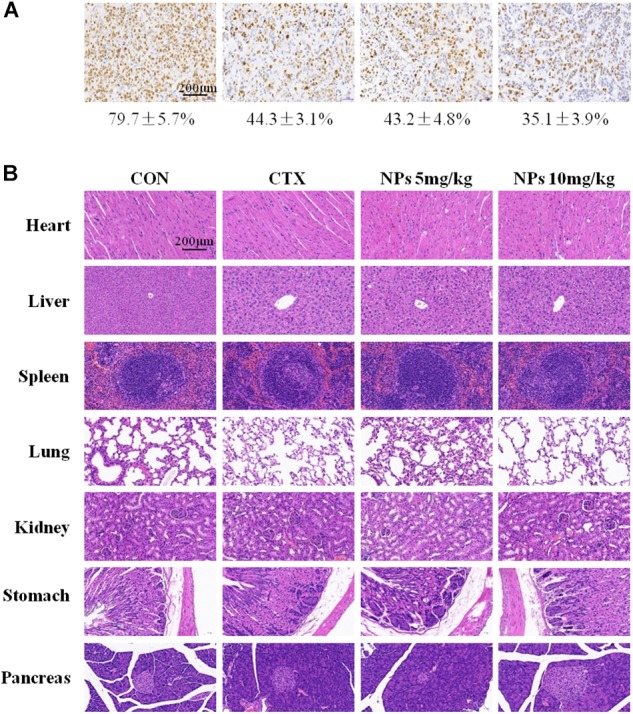
Immunohistochemical (IHC) analysis (IHC) analyses of tumors **(A)**, and histological analysis of seven principal organs excised from AsPC-1 tumor-bearing mice **(B)** in the control group, CTX-treated, NPs-treated (5 mg/kg), and NPs-treated (10 mg/kg) were performed as described in the Section “Materials and Methods.” Scale bar, 200 μm.

We also examined Ki67 expressions, a marker for cell proliferation (Figure 7A). There were fewer Ki67-positive cells in tumors treated with 5 mg/kg Zn-CuO NPs (43.2 ± 4.8%), compared with vehicle-treated tumors (79.7 ± 5.7%); and the group treated with 10 mg/kg Zn-CuO NPs resulted in even fewer Ki67-positive cells (35.1 ± 3.9%). In addition, the major organs of the mice (heart, liver, spleen, lung, kidney, stomach, and pancreas) in each group were collected for H&E staining, and the results showed that no obvious organ damage was found in all groups (Figure 7B), suggesting that Zn-CuO NPs displayed significant anti-tumor effect *in vivo* with low toxicity.

## Discussion

In the present study, we confirmed that Zn-CuO NPs was able to inhibit the growth of pancreatic cancer both *in vitro* and *in vivo*. Treatment with Zn-CuO NPs inhibited the tumor growth significantly in xenograft mice with low toxicity. The results provided primary evidence that the NPs had potential to be developed as novel anticancer agents for the treatment of pancreatic cancer.

It has been well established that autophagy plays an important role in cancer development and autophagy during tumor development in cancer therapy has been reported paradoxically to have roles in promoting both cell survival and cell death (Kondo et al., 2005; White and DiPaola, 2009; Rosenfeldt and Ryan, 2011). Over the years, a lot of metal oxide NPs have been reported to display anticancer activity by inducing autophagy. In most of the circumstances, elevated autophagy after nanomaterial treatment leads to increased cell death. Wu et al. confirmed that Fe@Au nanoparticles exhibited anticancer activity by mitochondria-mediated autophagy (Wu et al., 2011). However, there are a lot of nanoparticles inducing a protective properties for cancer cells. Silver NPs induced autophagic-promoted cell survival, and inhibition of autophagy by chemical inhibitors enhanced the silver NPs-elicited cancer cell killing (Lin et al., 2014). Recent studies by Zhang et al. also confirmed that the metal oxide nanoparticles nano-TiO2 induced a protect autophagy via antixodative mechanism (Zhang et al., 2016). Our present study revealed that the Zn-CuO NPs-induced autophagy resulted in enhanced anticancer activity. Our study provides more evidence that the autophagy induced by nanoparticles plays an important role in cancer development.

AMPK, as a negative regulator of mTOR signaling, regulates autophagosome formation (Yan et al., 2017). AMPK serves as a positive regulator of autophagy mainly by activating the phosphorylation of ULK1 (the ortholog of Atg1 in mammals) (Alers et al., 2012; Zhang P. et al., 2017). Narciclasine, an Amaryllidaceae isocarbostyril compound, induces autophagy-dependent apoptosis in triple-negative breast cancer cells by regulating the AMPK/ULK1 axis (Cao et al., 2018). Endosulfan, as an organochlorine pesticide, induces autophagy via the AMPK/mTOR signaling pathway triggered by oxidative stress (Zhang L. et al., 2017), while Panduratin A, a plant-derived active compound, induces protective autophagy in melanoma via the AMPK and mTOR pathway (Lai et al., 2018). The present results showed that Zn-CuO NPs induced autophagic cell death by activating the AMPK/mTOR/ULK1 pathway, as evidenced by the up-regulation of p-AMPK and p-ULK1, and the down-regulation of p-mTOR expression (Figure 4). Our studies suggested that targeting AMPK/ULK1 pathway may be a promising approach in the treatment of cancer.

## Conclusion

In conclusion, our results showed that Zn-CuO NPs inhibited the growth of pancreatic cancer both *in vitro* and *in vivo*. The induced autophagy by the nanoparticles is responsible for their anticancer effects and AMPK/mTOR pathway plays a vital role in Zn-CuO NPs induced cancer cell autophagy.

## Data Availability

All datasets generated for this study are included in the manuscript and/or the supplementary files.

## Author Contributions

XL, HX, and XKL designed the experiments. HX, CL, GQ, XHL, AF, and AG provided technical assistance. HX, CL, XL, and XHL assisted the investigators during the study. XKL provided consultation to the investigators on the study. XL carefully prepared the manuscript, and illustrations.

## Conflict of Interest Statement

The authors declare that the research was conducted in the absence of any commercial or financial relationships that could be construed as a potential conflict of interest.
